# Assessing control of postural stability in community-living older adults using performance-based limits of stability

**DOI:** 10.1186/1471-2318-8-8

**Published:** 2008-03-31

**Authors:** Myriam Jbabdi, Patrick Boissy, Mathieu Hamel

**Affiliations:** 1 Research Centre on Aging, Sherbrooke Geriatric University Institute, Sherbrooke, Quebec, Canada; 2 Université de Sherbrooke, Kinesiology Department, Faculty of Physical and Sports Education, Université de Sherbrooke, Sherbrooke, Quebec, Canada

## Abstract

**Background:**

Balance disability measurements routinely used to identify fall risks in frail populations have limited value in the early detection of postural stability deficits in community-living older adults. The objectives of the study were to 1) measure performance-based limits of stability (LOS) in community-living older adults and compare them to theoretical LOS computed from data proposed by the Balance Master^® ^system, 2) explore the feasibility of a new measurement approach based on the assessment of postural stability during weight-shifting tasks at performance-based LOS, 3) quantify intra-session performance variability during multiple trials using the performance-based LOS paradigm.

**Methods:**

Twenty-four healthy community-living older adults (10 men, 14 women) aged between 62 to 85 (mean age ± sd, 71.5 ± 6 yrs) participated in the study. Subjects' performance-based LOS were established by asking them to transfer their body weight as far as possible in three directions (forward, right and left) without changing their base of support. LOS were computed as the maximal excursion of the COP in each direction among three trials. Participants then performed two experimental tasks that consisted in controlling, with the assistance of visual feedback, their centre of pressure (COP) within two predefined targets set at 100% of their performance-based LOS. For each tasks 8 trials were performed. Ground reaction forces and torques during performance-based LOS evaluation and experimental tasks were recorded with a force plate. Sway area and medio-lateral mean COP displacement speed variables were extracted from force plate recordings.

**Results:**

Significant differences between theoretical LOS computed from maximum leaning angles derived from anthropometric characteristics and performance-based LOS were observed. Results showed that a motor learning effect was present as the participants optimized their weight-shifting strategy through the first three trials of each task using the visual biofeedback provided on their COP. Reliable measures of control of postural stability at performance-based LOS can be obtained after two additional trials after the learning phase (0.69 > ICC > 1.0).

**Conclusion:**

Establishing performance-based LOS instead of relying on estimations of theoretical LOS offers a more individualized and realistic insight on the true LOS of an individual. Performance-based LOS can be used as targets during weight-shifting postural tasks with real time visual feedback of the COP displacement to assess postural stability of community-living older adults. In order to obtain reliable results, a learning phase allowing subjects to learn how to control their COP displacement is needed.

## Background

Falling is one of the most serious health problems facing community-living older adults. Thirty to sixty percent of community-living older adults fall each year [1]. Consequences of falls result in considerable mortality, morbidity, reduced functioning and premature nursing home admissions [2]. Loss of balance is known to be one of the main intrinsic risk factors for falls in older adults [3,4]. Although clinical balance assessments (see [5] for references) such as the Berg Balance Scale, Tinetti Performance-Oriented Mobility Assessment, and Timed Up and Go Test are used by health professionals to evaluate balance deficits and fall risks among frail older adults, studies have shown that the use of these tests in healthy community-living older adults is limited by a ceiling effect and that these tests have limited value when trying to assess fall risks in this population [6,7]. Assessment of postural control and stability in healthy older adults through posturography is seen as a more promising and sensitive measurement approach for early detection or pre clinical changes in the postural control system [8,9]. In the last decade, commercially developed balance assessment systems (Chattecx^®^, Equitest^®^, Balance Master^®^) have been proposed as a way to obtain more precise, objective [10,11], and potentially more sensitive [12] measurements of subjects' postural stability. The Balance Master^®^, one of these turnkey measurement systems, combines centre of pressure (COP) measures with visual feedback on centre of mass (COM) displacements during postural control tasks. The assessment of postural stability at the subject's limits of stability (LOS) is one of these tasks and one of the evaluation options available in the Balance Master^® ^assessment modules. Postural stability at the LOS in the Balance Master^® ^is based on the assessment of sway parameters during weight-shifting to targets representing theoretical LOS and on the juxtaposition of visual feedback of the centre of body mass displacements with the projected targets. The theoretical LOS correspond to the maximum range in which the centre of body mass can be moved safely without changing the base of support [13]. This assumes that the body mass is part of a rigid segment and behaves as an inverted pendulum. Limits are represented by eight predefined targets posted on a screen and whose positions are calculated according to the maximum centre of body mass sway angles in the antero-posterior (A/P) and medio-lateral (M/L) directions [14].

While several studies have documented the psychometric qualities of the Balance Master^®^, to our knowledge no one has questioned the theoretical LOS used in its assessment module of postural stability or described the variability of the sway parameters obtained during repeated measures. Shumway-Cook [15] mentions that "stability limits are not fixed boundaries, but change according to the task." Several authors have used performance-based LOS, also called functional LOS, to assess postural stability of young and older subjects [16,17] and subjects with or without disease like Parkinson [18,19]. Postural stability decreases dramatically when measured in LOS conditions as much for young people as for older adults with or without balance disabilities. Considering the results of these studies, and accepting that assessing postural stability at performance-based LOS would represent a challenge closer to reality, we propose the use of a postural control measurement paradigm at performance-based LOS rather than theoretical limits to assess postural stability. The objectives of this study were to 1) measure performance-based LOS in community-living older adults and compare them to theoretical LOS computed from data proposed by the Balance Master^® ^system, 2) assess the feasibility of evaluating postural stability at performance-based LOS in this population, and 3) quantify intra-session performance variability during multiple trials using the performance-based LOS paradigm.

## Methods

### Participants

Twenty-four healthy community-living older adults (10 men, 14 women) aged between 62 to 85 (mean age ± sd, 71.5 ± 6 yrs) participated in the study. The inclusion criteria for participation were: age (60 and older), being able to stand in an upright position without assistance for 60 sec., living in the community, absence of cognitive and neurological impairments, and absence of visual deficits such as daltonism or poor visual acuity. Data were collected over a 4-month period at the Research Centre on Aging, Sherbrooke, Quebec, Canada. The Sherbrooke Geriatric University Institute Institutional Review Board approved this investigation and all participants gave their informed consent. Prior to testing, the participants were evaluated on a battery of health questionnaire and functional tests including: the short form health survey (SF-36), the Berg balance test, and the forward functional reach test [5].

### Experimental procedure and equipment

#### Assessment of limits of stability

The measurement approach used in this study is based on the assessment of postural stability during weight-shifting tasks at LOS established a priori during performance-based tasks. Subjects' performance-based LOS were established by asking them to transfer their body weight as far as possible in three directions (forward, right and left) without changing their base of support. For feasibility and security reasons we did not assess the performance-based LOS in the backward direction. Moreover, a study demonstrated that subjects were able to shift their COP less further in posterior than in anterior, left or right directions [17]. Participants were asked to use an ankle strategy rather than a hip strategy when weight shifting to execute the tasks. Performance-based LOS were recorded barefoot, three times in each direction. The participants were asked to hold the leaning position until stability was achieved (about few seconds). Measurements were recorded when stability was reached and LOS were computed as the maximal excursion of the COP in each direction among the three trials. No feedback was provided to the subjects during LOS evaluation. The subjects' theoretical LOS were estimated according to their centre of gravity (COM) height and maximum body sway leaning angles as described in [14]. Maximum body sway angles are based on the assumptions that the centre of body mass in a stable upright position is located at 2.3 angular degrees ahead of the true frontal plane, approximately at half of the person's height (COM = 0.5527 × height), and that its displacements are constrained inside an inverted cone, with theoretical LOS determined as extending 6.25° in the anterior plane, 4.45° in the posterior plane, and 8° to each side [14]. Obviously, these assumptions are very influenced by the anthropometric characteristics of the subject. In our study, theoretical LOS in cm were computed using the following equations: Forward LOS = COMheight * TAN (6.25°) and Right and Left LOS = COMheight * TAN (8°). For example, an individual 160 cm tall will have a COM positioned 88.4 cm from the ground and, according to maximum leaning angles, his/her forward theoretical LOS will be 9.68 cm and 12.43 cm for his/her right and left theoretical LOS.

#### Control of postural stability at limits of stability

**Figure 1:**
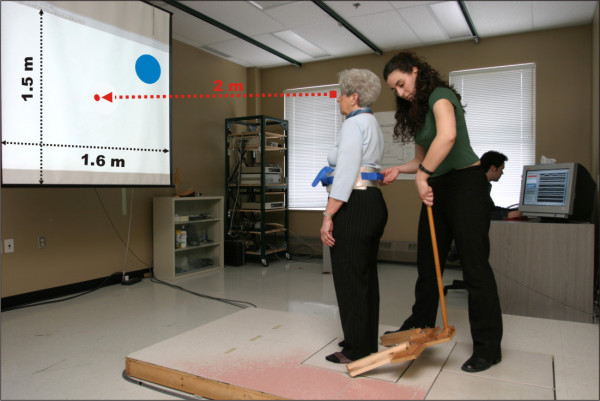
I**llustration of experimental paradigm and measurement set-up.**

After recording the performance-based LOS, two experimental tasks were presented to the subjects and consisted in weight-shifting and controlling COP displacements within right lateral and right antero-lateral targets. The right lateral target was placed at 100% of the subject's performance-based LOS in the right direction, whereas the right antero-lateral target was placed at an angle of 45° between forward and right direction directly on an ellipse passing by the 100% performance-based LOS of the subject in the forward and in the right direction. Real time visual feedback of the COP displacement was given to the subject to guide task execution (Figure 1). The feedback was necessary to insure that subjects shifted their weight at their maximum LOS and tried to maintain their stability within a predefined region when the target was reached. The target tolerance (number of pixels around a COP position on the screen corresponding to a given % of LOS) was scaled for all subject to 10%. The visual feedback provided for the target area changed from blue to green when the subject was not able to stay within (i.e. COP outside of the target). Prior to each experimental task assessing postural stability at performance-based LOS, a familiarization period with the visual biofeedback of the COP displacement was given to the participants. The duration of the familiarization with the visual feedback was a function of the subject's understanding of the relationship between weight shifting strategies and the corresponding displacement of the COP representation in space. This was variable from one subject to the other but took less than 5 minutes in all the subjects. This period lasted until the evaluator was sure the participant understood how the visual biofeedback functioned. Upon completion of the familiarization phase, the following instruction was given to the subject for the execution of the experimental tasks: "You have to reach the blue target with the red dot, as fast as possible, by transferring your weight in the direction of the target and then stay as long as possible within the target, or if this is not possible, stay as near to the target as possible. The blue target will change to a green target when you succeed." No subject failed to control their equilibrium in these tasks after familiarization was over.

Ground reaction forces and torques during performance-based LOS evaluation and experimental tasks were recorded with an AMTI OR6-7 force plate. Orthogonal forces and torques were sampled at 2000 Hz and converted using a 16-bit acquisition card (DAQPad-6052E, National Instruments) connected to a PC. A dedicated data collection program with a Labview visualization interface was used to compute COP position in real time and provide feedback on COP position with respect to predefined weight-shifting targets. The sampling rate of force plate signals was 2000Hz. COP position computations from force plate signals were done on blocs of 300 data points at 100Hz and then displayed on a screen with a refresh rate of 60Hz. The latency of the feedback on COP position was about 150 ms. A BenQ SL705X XGA DLP projector was used to project COP biofeedback and predefined weight-shifting targets on a white screen (150 cm × 160 cm) which was placed two meters in front of the participant. Finally, a foot template was used to standardize the position of the feet between each trial. The space between the heels was 12 cm and the angle made by each foot with the saggital plane was 15°. Target positions on the visualization screen for the experimental tasks were set at fixed positions so that the target for the subject's LOS corresponded to the maximum width and length of the screen (1024 × 768 pixels). The biofeedback on the COP trajectory with respect to the target was normalized (expressed as a displacement in mm per pixel) in relation to each subject's performance-based LOS in the M/L and A/P directions and the resolution of the visualization screen. Each experimental task was composed of 8 trials lasting 60 seconds each. Participants were asked if they wished to rest between each trial to limit fatigue and stress effects. The first experimental task consisted in transferring the COP (represented by the red dot) at the performance-based LOS in the right lateral direction and maintaining the COP within the predefined target as long as possible up to 60 sec. For the second task, the orientation of the movement changed. The participant had to move in the right antero-lateral direction to reach the target. The order of the tasks was fixed across subjects.

### Measures of postural stability and statistical analysis

COP in A/P and M/L planes of motion during each trial of both experimental tasks were computed from filtered force plate signals (2^nd ^order low-pass Butterworth filter with a 50 Hz cut-off frequency). Mean COP displacement speeds (cm/sec) and COP sway areas (cm^2^) were extracted from each trial using methods proposed by Oliveira [20]. Descriptive statistics and paired T-tests were performed to compare theoretical LOS computations and performance-based LOS measurements. A paired T-Test was performed to compare the lateral weight-shifting task to the antero-lateral weight-shifting one. ANOVAs for repeated measures were performed on postural control variables extracted from trials (n = 8) in both tasks. A posteriori contrast analyses were performed to identify differences between trials and determine a stabilization phase (i.e. first instance where no significant difference between two consecutive trials was found). Intra-class correlation coefficients (ICC) (two-way random model with an absolute agreement type) were computed on two consecutive measures during the stabilization phase, and the predicted reliability of the measures with respect to a given number of trials was assessed using the Spearman-Brown formula. The statistical significance threshold was set at p ≤ 0.05 and confidence intervals (CI) for ICC at 95%. All statistical analyses were performed with SPSS 12.0 software.

## Results

### Participants

The participants' self-perceived general health was good, with 22 participants out of 24 scoring higher than the 75 percentile on the general health dimension of the SF-36. They also demonstrated close to perfect functional balance on the Berg balance scale (mean = 51.3/56 points; SD = 6.5 points) with only 7 out the 24 participants showing balance problems placing them at risk of falling (score of ≤ 49/56 points on the scale) (see [21] for cut-off values). Scores on the forward functional reach test were on average higher (mean = 28.09 cm; SD = 7.62 cm) than the proposed cut-off score associated with a risk of falling [22], with 9 participants scoring below 25.4 cm. Body Mass Index (kg/m^2^) values of participants varied from normal (5 subjects/24), to overweight (12 subjects/24) and obese class 1 (12 subjects/24), see Table 1.

**Table 1:** Characteristics of the participants.

	**Women (n = 14)**	**Men (n = 10)**	**Group (n = 24)**
**Age (yr)**	71.8 ± 6.7	71.1 ± 5.3	71.5 ± 6.03
**Weight (kg)**	67.3 ± 10.8	86.9 ± 8.8	74.4 ± 13.5
**Height (cm)**	157.7 ± 7.5	169.1 ± 7.2	162.4 ± 9.1
**SF-36 General health scale (percentile)**	96.2 ± 7.4	76.39 ± 40.9	89 ± 26.2
**Berg Balance test (/56 points)**	51.8 ± 5.8	50.6 ± 7.6	51.3 ± 6.5
**Forward Functional reach (cm)**	28.8 ± 6.6	25.6 ± 9.1	28.9 ± 7.6

### Theoretical and performance-based LOS

**Figure 2:**
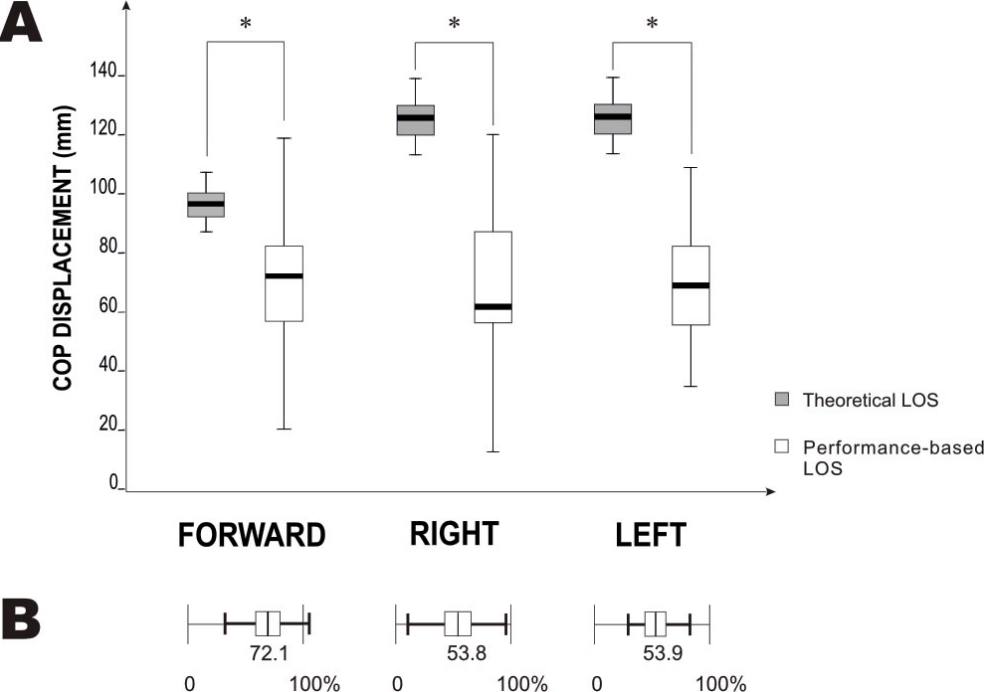
**Comparison of theoretical and performance-based limits of stability (LOS)**. **A) **Box plot illustrating medians and standard deviations for theoretical and performance-based LOS in the forward and right and left lateral directions for 24 older adults. **B) **Box plot illustrating differences between theoretical and performance-based LOS (expressed as % of theoretical LOS). Means and standard deviations are presented. Statistically significant differences are indicated with * p < 0.001.

Values for LOS computed from theoretical maximum leaning angles and performance-based measures for 24 participants across leaning directions are shown in Figure 2. Inter-subject variability, as seen by whisker ranges, is generally greater for the performance-based than the theoretical LOS. Paired T-test results revealed significant differences between theoretical and performance-based LOS in all of the leaning directions (p < .001). Results also show that for all of the directions, theoretical LOS were larger than performance-based LOS. Participants were able to reach only 72% of the mean theoretical LOS in the forward leaning direction and about 54% in both lateral leaning directions.

### Postural stability control at performance-based LOS and trial variations

**Figure 3:**
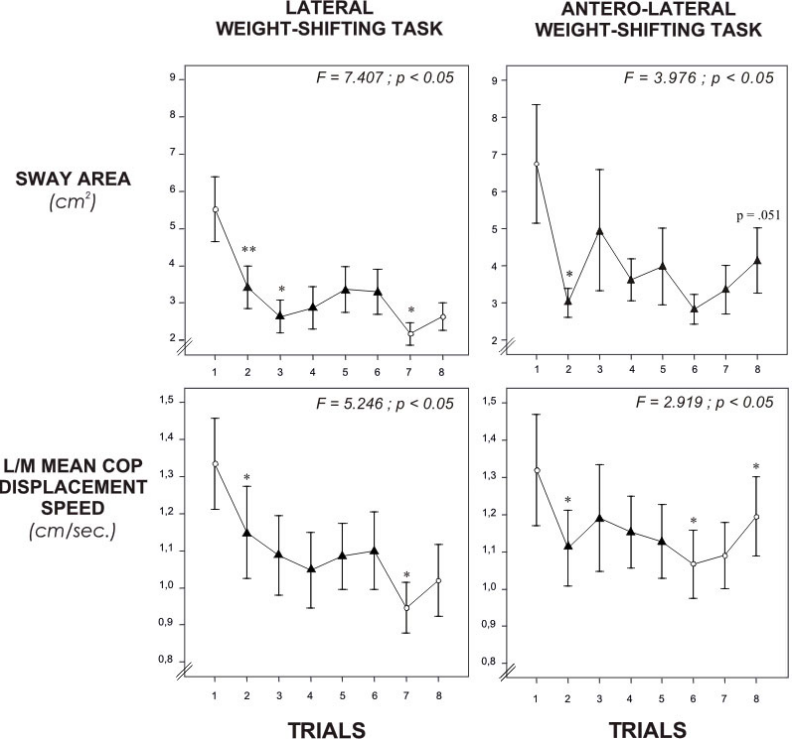
**Postural control measures obtained during weight-shifting within right lateral and right antero-lateral predefined targets placed at 100% of the subject's limits of stability**. Results represent group means and standard deviations (n = 24 participants) for each trial. Triangle symbols indicate improvement and stabilization phases of the performance in the 8 trials. Statistically significant differences are indicated with * p < 0.05 and ** p < 0.001.

Postural stability control variables (sway area and M/L mean COP displacement speed) from 8 trials at performance-based LOS during lateral and antero-lateral weight-shifting tasks are presented in Figure 3. Paired T-test analyses between tasks show no significant differences between the two tasks for both variables (p = .181 for the sway area; p = .319 for the M/L mean COP displacement speed). Even though the order of the tasks was fixed, it probably did not have any impact on the results considering that the two tasks are equivalent in terms of difficulties.

However, results from ANOVAs with repeated measures show the presence of a trial main effect (p < 0.05). This trial main effect was consistent across both tasks and both variables, suggesting a learning of the weight-shifting tasks. More specifically, results from contrast analyses show the presence of an improvement phase between the first and second trials for the sway area variable during the antero-lateral weight-shifting task (p < 0.001) and for the M/L mean COP displacement speed variable during the lateral and antero-lateral weight-shifting task (p < 0.05). With respect to the sway area variable during the lateral weight-shifting task, the improvement phase extended to the third trial. Overall, after the third trial, postural control performances stabilize across the rest of the trials with the exception of some statistical differences observed between the 5^th ^and 6^th ^or 6^th ^and 7^th ^trials for certain variables and tasks.

**Figure 4:**
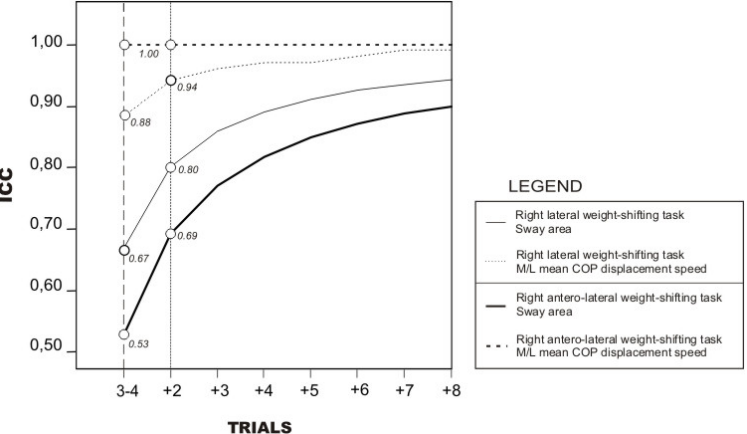
**Optimal number of trials needed after learning phase to get reliable postural control measures during weight-shifting within right lateral and right antero-lateral predefined targets placed at 100% of the subject's limits of stability**. Reliable measures on both postural control parameters can be achieved after the learning phase (3 trials) by having the participant perform 2 additional trials. The Y axis show the ICC values. The X axis present the number of additional trials added to the mean of the 3^rd ^and 4^th ^trials. For exemple, the ICC value obtained for the right antero-lateral weight-shifting task (sway area variable) is about 0.53 for the mean of the 3rd and 4th trials. If we add two trials, then we realize 5 trials, the fidelity of the measurement increase up to 0.69.

ICC estimates calculated using the Spearman-Brown formula for M/L COP displacement speed and sway area variables obtained during the experimental tasks are presented in Figure 4. ICCs were computed for each variable and each task from measures obtained during the third and fourth trials (stabilization phase). Results showed that initial ICCs obtained after the stabilization phase were respectively .53 (0.18 > CI_95% _> 0.76) in the antero-lateral and .67 (0.37 > CI_95% _> 0.84) in the lateral weight-shifting tasks for the sway area variable. For the M/L mean COP displacement speed variable, the ICCs obtained for one measure were higher than for the previous variable and were .88 (0.74 > CI_95% _> 0.95) in the lateral and 1.0 (0.99 > CI_95% _> 1.00) in the antero-lateral weight-shifting tasks. While increasing the number of trials after the stabilization phase automatically increases the reliability of the measure, reliable performances on both parameters can be achieved by having the participant perform two additional measurement trials after the learning phase (3 trials). Results show that with a total of five trials, the measurement approach would reach a moderate to excellent reliability level (0.69 to 1.0).

## Discussion

### Theoretical vs. performance-based limits of stability

We investigated the validity of assessing control of postural stability at limits of stability established during performance-based LOS tasks in community-living older adults. LOS established during weight-shifting tasks suggest that community-living older adults cannot reach the theoretical LOS determined from maximum leaning angles proposed by NeuroCom International Inc. and used in the assessment module of the Balance Master^®^. Our results suggest that theoretical LOS computed from maximum leaning angles significantly overestimate the capacity of community-living older adults to shift their body weight as far as possible from an upright standing position without changing their base of support. As maximum leaning angles were established on healthy adults without consideration for age [14], it appears that these maximum leaning angles cannot be transposed to community-living older adults. This can also be said for performance-based LOS as significant differences, independently of the participants' anthropometric characteristics, were found between young and older individuals [23].

In our study, community-living older adults were able to reach about 72% of their theoretical LOS in a forward direction and approximately 54% in lateral directions. These results are significantly lower than those presented by NeuroCom International Inc. to support the use of theoretical LOS based on maximum leaning angles in older adults [14]. They assessed the "maximum voluntary excursion" of 55 older adults 60 to 79 years of age and reported that these participants were able to reach 92% and 98% of their theoretical LOS computed from maximum leaning angles in the forward and lateral directions respectively. The discrepancy between their results and ours could hypothetically be due to differences in the profiles of the participants (e.g. functional balance capacity, anthropometric characteristics, fitness level, age, motivation,...). However, with the exception of two of the participants, most of the individuals in our study showed good functional balance on the clinical scales used and the participants' self-perceived health was good. Differences in age and fitness level could also have played a role, but since no information on the individual characteristics of the subjects in the NeuroCom International Inc. study is provided, the interpretation of such a discrepancy on that basis is not possible. Anthropometric characteristics of subjects have been shown to affect the estimation of the COM. They probably played a role in the differences observed. Finally, motivation and perceived capability could also have a great influence in the capacity of the subject to shift their COP toward the extremes [17]

Control over the movement strategies used by the subjects to reach their LOS may also have affected LOS values to some extent. As the theoretical LOS are computed using maximum leaning angles based on the model of a rigid segment behaving like an inverted pendulum, it can be assumed that the strategy used by the subjects to shift their weight to their limits of stability during performance-based LOS, especially in respect to trunk motion, could influence LOS values. For example, in our study, we asked the participants to lean slowly to reach their LOS using an ankle strategy and then maintain the position for a few seconds before returning to the initial stable position. If participants used trunk flexion or compensated by protracting their shoulders, the trial was stopped, the instructions were repeated and the subject was asked to perform a new weight-shifting trial. In the Balance Master^® ^protocol, individuals are asked to lean as quickly as possible within 8 seconds to reach targets representing their theoretical LOS and no specific instructions or control regarding the strategy used is provided. In further studies, detailed attention should be given to the way in which the LOS evaluations are carried out and kinematic analysis should be used to characterize control strategies.

### Control of postural stability at performance-based LOS

One of the objectives of this study was to quantify intra-session performance variability in the control of postural stability with visual biofeedback during multiple trials at performance-based LOS. Results showed that a motor learning effect was present as the participants optimized their weight-shifting strategy through the first three trials of each task according to the visual biofeedback provided regarding their COP. As the ability to perform new motor skills changes with experience and practice [24], the proposed tasks underlying this measurement paradigm appear to be relatively simple for community-living older adults to learn. Their postural stability performances reached a stable baseline after three repeated trials. In this study, subjects performed eight trials lasting 60 seconds each per weight-shifting task. Considering that performing fewer trials decreases the time of data acquisition and the cumulative physical and mental fatigue effect associated with the repetition of many trials, Spearman-Brown ICC results showed that reliable performances in terms of postural stability control at performance-based LOS can be obtained with two trials after the learning phase. However in both experimental tasks, mean COP displacement speeds in the M/L and A/P directions seem to be more reliable than the sway area variable. A higher muscular stifness may also affect the results [25]. However, since no EMG data were collected, it is difficult for us to comment on this point.

Finally, it should be noted that certain limitations in the study design confine the scope of the interpretation of the results presented. Postural stability control variables (sway area and M/L mean COP displacement speed) were measured during performance-based LOS during lateral and antero-lateral weight-shifting tasks. We computed numerous traditional measures of stability from the COP data. For the sake of simplicity and considering that we were not trying to address the validity of the measurements with such a small sample, we choose to present reliability results for two of the most used parameters of stability (sway area and M/L mean COP displacement speed). It would have been interesting to use other approaches, such as stabilogramm diffusion analyses, to characterize the dynamic of COP and to better understand postural stability of the subjects of our sample [20]. While the tasks performed at performance-based LOS were chosen on the basis of two factors: 1) making sure that we had a direction that combine both mediolateral and anterior weight shifting as COP displacements in the mediolateral direction are more difficult and associated with risk of falls, 2) choosing a direction similar to existing balance tests (i.e. functional reach), these directions represent only a small subset in the range of LOS. Furthermore, both targets proposed were on the right side of the subjects, regardless of their preferred side.

## Conclusion

Limits of stability established during weight-shifting tasks suggest that community-living older adults cannot reach theoretical LOS determined from maximum leaning angles. Control of postural stability through visual feedback of COP at LOS established during performance-based tasks showed that community-living older adults can easily learn the experimental tasks used in the proposed measurement approach after three repeated trials. Reliable measures can be obtained after two subsequent trials. However, the small sample size of this study limits the generalizability of these results. Further studies will investigate the clinical value of this experimental paradigm and the sensitivity of posturography measures derived from such tasks to discriminate between individuals with different degrees of balance impairment.

## Competing interests

The author(s) declare that they have no competing interests.

## Authors' contributions

MJ participated in the design of the study, carried out the acquisition, analysis and interpretation of the data, drafted and revised the manuscript. PB conceived the design of the study, coordinated the development of the experimental paradigm and the study, supervised the data analysis, drafted and revised the manuscript. MH contributed to the development of the experimental paradigm and revised the manuscript. All authors read and approved the final manuscript.

## Pre-publication history

The pre-publication history for this paper can be accessed here:


